# Clinical features associated with immune checkpoint inhibitor nephritis: a single-center clinical case series

**DOI:** 10.1007/s00262-024-03775-6

**Published:** 2024-08-06

**Authors:** Ramya Muddasani, Neel Talwar, Isa Mambetsariev, Jeremy Fricke, Mercury Lin, Daniel Schmolze, Andrew Yue, Amna Rizvi, Ravi Salgia

**Affiliations:** 1 Department of Medical Oncology and Therapeutics Research, City of Hope Comprehensive Cancer Center, 1500 East Duarte Road, Duarte, CA 91010 USA; 2 Department of Pathology, Cedars-Sinai Medical Center, Los Angeles, CA USA; 3 Department of Pathology, City of Hope Comprehensive Cancer Center, Duarte, CA USA; 4 Department of Nephrology, City of Hope Comprehensive Cancer Center, Duarte, CA USA

**Keywords:** Checkpoint, Immunotherapy, Nephritis, Pembrolizumab, Nivolumab, AKI

## Abstract

**Background:**

Acute kidney injury (AKI) has been well described as a complication of immune checkpoint inhibitor therapy. We present a series of patients, the majority with lung adenocarcinoma, who developed AKI while actively receiving immune checkpoint inhibitors.

**Methods:**

This is a retrospectively analyzed clinical case series of six patients treated at City of Hope Comprehensive Cancer Center. Data were collected on gender, age, ethnicity, comorbidities, concomitant medications, type of malignancy, treatments, and renal function. All patients underwent renal biopsy for classification of the mechanism of AKI. Comprehensive genomic profiling (CGP) was performed on tumor tissue for all patients.

**Results:**

Patterns of AKI included acute interstitial nephritis and acute tubular necrosis. Contributing factors included the use of concomitant medications known to contribute to AKI. All but two patients had full resolution of the AKI with the use of steroids. There were several mutations found on CGP that was notable including an Exon 20 insertion as well as multiple NF1 and TP53 mutations. There was high PD-L1 expression on tumor tissue noted in two out of six patients. In addition to AKI, a subset of patients had proteinuria with biopsies revealing corresponding glomerular lesions of minimal change disease and focal and segmental glomerulosclerosis.

**Conclusions:**

Our case series demonstrates that AKI from immune checkpoint inhibitors has a variable presentation that may require an individualized treatment approach. Further studies are needed to identify biomarkers that may help identify those at risk and guide the management of this condition.

## Introduction

Immune checkpoint inhibitors (ICIs) have emerged as a practice-changing treatment approach for patients with advanced solid tumor malignancies, but serious immune-related side effects (IRAEs) can occur which may hinder the ability to continue treatment in a subset of patients. An increasingly recognized IRAE is ICI-induced renal failure [1–3]. ICIs function by inhibiting the immune system’s breakpoints, leading to immune activation against cancer cells. However, this immune activation can also lead to unwanted side effects due to inflammation of healthy tissues. In this scenario, the immune system attacks the kidneys, which can lead to renal failure which can manifest in various histopathologic fashions some of which include acute interstitial nephritis, acute tubular necrosis (ATN), glomerulonephritis, and renal vascular toxicity [4]. These conditions can impair the renal’s ability to filter, maintain electrolyte balance, and regulate fluid levels in the body. Consequently, patients may experience symptoms of renal failure including decreased urine output, swelling, fatigue, electrolyte imbalances, and elevated creatinine levels. Understanding the mechanisms underlying ICI-induced renal failure is essential for early recognition and appropriate management of this adverse event.

Although ICI-induced renal failure is a concerning side effect, it is important to note that only a small percentage of those who receive immunotherapy will experience renal toxicity [5, 6]. Risk factors for developing renal complications are still being investigated, including pre-existing renal conditions, concomitant use of other medications, and individual patient characteristics. Additionally, there are limited data illustrating a higher incidence of ICI-induced renal failure in patients with melanoma and non-small cell lung cancer patients as compared to other solid tumors. This may be related to the more durable responses to immunotherapy seen in these tumor types as compared to other solid tumors [7]. Ongoing research aims to identify predictive biomarkers and refine treatment protocols to minimize the occurrence of renal toxicity and improve patient safety.

Recognizing and managing ICI-induced renal failure are crucial for providing comprehensive care to cancer patients receiving immunotherapy. Our case series aims to advance our understanding of the risk factors associated with ICI-induced renal failure such that we can enhance patient safety and optimize treatment outcomes. In this study, we discuss a series of cases including patients with various solid malignancies treated with immunotherapy who subsequently developed IO-induced acute kidney injury (AKI).

## Materials and methods

This is a retrospective study of patients with solid organ malignancies treated with ICIs at City of Hope Comprehensive Cancer Center. Data were collected on fourteen patients with AKI who had received ICIs from August 2017 to December 2022. Six of the 14 underwent renal biopsies due to suspected ICI-induced renal failure. The selection of patients warranting further workup with a renal biopsy was based on the clinician’s discretion and was typically conducted in the setting of rapidly progressive renal failure with no clear alternative etiology. Subsequent data collection included gender, age, ethnicity, comorbidities, concomitant medications [with an emphasis on medications known to contribute to AKI i.e., non-steroidal anti-inflammatory drugs (NSAIDs), proton pump inhibitors (PPIs), antibiotics, and angiotensin-converting enzyme inhibitors and angiotensin II receptor blockers], type of malignancy, series of oncologic treatments, baseline renal function, stage of AKI upon presentation, and time to resolution. All procedures performed in this study were in accordance with the ethical standards of the City of Hope Institutional Review Board (IRB) as per IRB #20289 and the Declaration of Helsinki (as revised in 2013). The City of Hope IRB granted a waiver of informed consent under 45 CFR § 46.116 based on determination that this research meets the following requirements: (i) The research involves no more than minimal risk to the subjects; (ii) the research could not practicably be carried out without the requested waiver; and (iii) the waiver will not adversely affect the rights and welfare of the subjects.

All patients included underwent renal biopsies which were analyzed by the Renal Pathology division at Cedars-Sinai Medical Center, Department of Pathology and Laboratory Medicine. Biopsy cores were obtained under ultrasound guidance using a 16-gauge needle. Renal biopsies were evaluated by subspeciality fellowship-trained and board-certified renal pathologists and studies included light microscopy (hematoxylin and eosin, Jones’ silver, periodic acid-Schiff, and Masson’s trichrome stained sections), immunofluorescence microscopy (stained with fluoresceinated antisera to human IgG, IgA, IgM, C1q, C3, albumin, fibrin, and kappa and lambda immunoglobulin light chains), and ultrastructural examination by electron microscopy. The degree of tubulointerstitial scarring in the renal cortex was graded as minimal (less than 10%), mild (10 to 25%), moderate (26 to 50%), and severe (more than 50%).

## Results

### Demographics/clinical characteristics

The male-to-female ratio was 1:6, and the median age was 65 years. Five out of six patients (83%) had concomitant PPI use, five out of six (83%) patients were receiving antibiotics, and two out of six (33%) were on NSAIDs. Five of the six (83%) patients in our series were diagnosed with metastatic lung cancer, with the remaining patient (17%) diagnosed with endometrial adenocarcinoma. Two patients (33%) had previously received other types of oncological treatment without remission. For four patients (67%), ICIs represented the first line of oncologic treatment. The ICI therapies used were: pembrolizumab monotherapy (33%), pembrolizumab in combination with chemotherapy or TKI (50%), and dual-checkpoint inhibitor therapy with ipilimumab and nivolumab (17%).

### Acute kidney injury

Five out of six patients (83%) had a normal baseline renal function. The time between the ICI administration and the presentation of renal disease ranged from 2 weeks to 11 months with a median onset of 2 months. We assessed renal injury using the acute kidney injury network criteria (AKIN). Three patients (50%) met AKIN stage 3 criteria and the others presented with stage 1 (17%) and stage 2 (33%). There were various patterns of renal injury noted. Four out of six patients (67%) had tubulointerstitial nephritis with concomitant acute tubular necrosis (ATN), one out of six patients (17%) had acute tubular injury (ATI)/ATN and minimal change disease (MCD), and one out of six patients (17%) had acute tubular injury and focal and segmental glomerulosclerosis (Table 1).

**Table 1:** Type of renal injury, treatment of ICI-related AKI, cancer type and comprehensive genomic profiling

Case	Type of renal injury	Treatment when AKI developed	Line of therapy	Concomitant medication use	AKIN stage	Initial treatment of AKI	Renal recovery
1	Acute tubulointerstitial nephritis, ATN	Pembrolizumab/Pemetrexed	1st line	Lisinopril, Moxifloxacin	3	Pulse dose prednisone (500 mg daily)	Complete
2	Acute tubulointerstitial nephritis, ATN	Carboplatin/Pemetrexed/Pembrolizumab	1st line	Acetaminophen, Pantoprazole	2	Prednisone (0.5 mg/kg	Complete
3	ATI/ATN, FSGS	Pembrolizumab/Lenvima	3rd line	Aspirin, Omeprazole	1	Prednisone (1 mg/kg)	Complete
4	ATN, Focal acute interstitial nephritis, mild arteriosclerosis	Pembrolizumab	1st line	Acetaminophen, Ibuprofen, Pantoprazole, Levofloxacin	3	Methylprednisolone (1 mg/kg)	None
5	Acute tubulointerstitial nephritis, mild arteriosclerosis	Pembrolizumab	1st line	Levofloxacin, Pantoprazole	3	Prednisone (1 mg/kg)	Complete
6	ATN, MCD, mild arteriosclerosis	Nivolumab/Ipilimumab	3rd line	Amlodipine, Clindamycin, Glimepiride, Losartan, Repaglinide, Sitagliptin	2	Tacrolimus (2 mg PO BID)	Partial

Case	Age at diagnosis	Gender	Cancer type	Molecular test	PD-L1 expression	TMB	MSS status	Mutations present
1	71	F	Lung adenocarcinoma	Foundation one	0%	Intermediate(10 Muts/Mb)	Stable	BRCA2 Deletion Exon 13–25, MED12 Q2119_Q2120insHQQQ, NF1 M968_K969 > I*, RB1 R7fs*24, STK11 M1?, TP53 P152fs*18
2	60	F	Lung adenocarcinoma	HopeSeq	0%	Unknown	Stable	FBXW7 T442Cfs*53, KRAS G12D
3	49	F	Endometrial adenocarcinoma, endometrioid type	Foundation one	0%	Low (4 Muts/Mb)	Stable	ARID1A S1587fs*25, ATRX P525fs*11, MLL R2830*, NOTCH3 G594fs*12, TP53 R175H
4	69	M	Lung adenocarcinoma	HopeSeq	80%	Unknown	Stable	SETD2 S188*, SMAD4 Q366*
5	61	F	Lung adenocarcinoma	Caris	90%	Unknown	Stable	EP300 Q259, PBRM1 G1447*, RET C620*, TP53 R158L
6	74	F	Lung adenocarcinoma	Foundation one	5%	Low (4 Muts/Mb)	Stable	CDKN1A R86W, EGFR N771_P772insV (Exon 20 insertion), ERBB4 amplification, FGFR4 amplification, NFKBIA amplification, NKX2-1 amplification, TERC amplification, Splice Site 783-31_795del44

### Treatment of renal injury

For five of the six patients (83%), the initial line of treatment for ICI-induced AKI included steroids of some form. However, one patient (17%; case 6) diagnosed with renal injury in the form of ATN and MCD was unable to tolerate steroids due to a prior history of steroid-induced psychosis as such she was started directly on tacrolimus. For the patient (case 3) with FSGS and ATI/ATN, it is important to note that steroid therapy was initiated approximately 1 week prior to the biopsy. Of these patients, four (66%) had a full recovery, while one patient (17%) had a partial recovery, and one patient (17%) required ongoing hemodialysis. All patients required cessation of ICI treatment after the diagnosis of AKI and none were rechallenged.

### Comprehensive genomic profiling

All six patients had comprehensive genomic profiling (CGP) performed on pathologic tumor specimens. Of these six patients, one (17%) presented with an actionable alteration specifically an EGFR exon 20 insertion. Three out of six patients (50%) harbored TP53 mutations in their tumor and two out of six patients (33%) exhibited NF1 mutations. Two out of six (33%) of patients had high PD-L1 expression (> 50%). Tumor mutational burden (TMB) was evaluable in three out of six patients. Two out of three patients (67%) had low TMB while one out of three had intermediate TMB (33%). Microsatellite stability status (MSS) was available in all patients, and all patients were noted to have tumors that were MSI-low. There were several other comutations present on CGP, which have been summarized in Table 1.

### Case summaries

#### Case #1

**Fig. 1:**
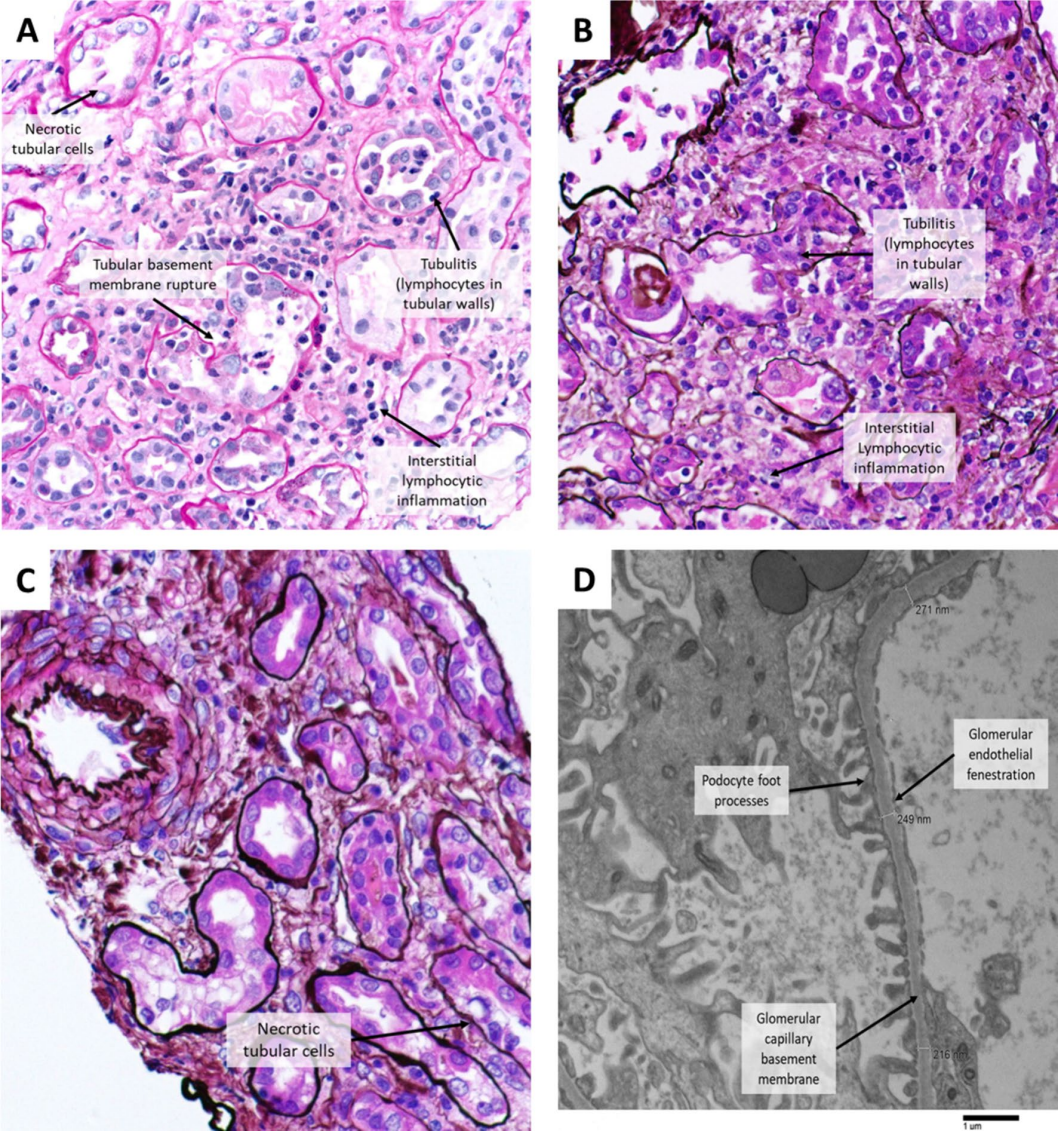
**A** Case 1: Acute tubulointerstitial nephritis showing interstitial inflammatory cell infiltrate, edema, and tubulitis (inflammation in tubular walls). There is also acute tubular necrosis. PAS stained, 400X magnification. **B** and **C** Case 2: Acute tubulointerstitial nephritis and acute tubular necrosis. The sampled artery shows no evidence of thrombotic microangiopathy. Jones’s Silver stain, 400X magnification. **D** Case 2: Incidental detection of segmental glomerular capillary basement membrane thinning. Electron microscopy, 6000X magnification

A 71-year-old female with stage IV adenocarcinoma of the lung started on carboplatin/pemetrexed/pembrolizumab developed stage 3 AKIN 11 months after treatment at which time the patient was on pemetrexed/pembrolizumab maintenance. Baseline creatinine was 0.75 mg/dL which increased to 4.35 mg/dL. Notable concurrent medications included lisinopril and antibiotics. A renal biopsy showed active tubulointerstitial nephritis, acute tubular necrosis, and arterial nephrosclerosis. Glomerular disease and thrombotic microangiopathy were not identified. There was mild tubulointerstitial scarring (Fig. 1). Notable concurrent medications included lisinopril and antibiotics. The AKI was treated with 500 mg IV methylprednisolone daily over 3 days followed by 60 mg IV daily. The patient also required a short course of hemodialysis followed by a 2-week course of high-dose steroids with improvement in renal function. She was subsequently placed on a prednisone taper with full resolution of renal injury within 3 months. ICI therapy was permanently discontinued due to the degree of renal injury.

#### Case #2

A 60-year-old female with stage IV adenocarcinoma of the lung started on carboplatin/pemetrexed/pembrolizumab developed stage 2 AKIN 4 months after treatment at which time she was on maintenance pemetrexed/pembrolizumab. Other notable factors include concomitant pantoprazole usage. Her baseline creatinine was 0.61 mg/dL which increased to a peak of 2.92 mg/dL. Renal biopsy revealed acute tubulointerstitial nephritis and acute tubular necrosis. There was also an incidental detection of segmentally thinned glomerular capillary basement membranes. Thrombotic microangiopathy was not identified. There was minimal tubulointerstitial scarring (Fig. 1). AKI was treated with prednisone 40 mg and started upon diagnosis with a subsequent slow taper with full resolution after 2 months. ICI therapy was permanently discontinued given renal injury.

#### Case #3

**Fig. 2:**
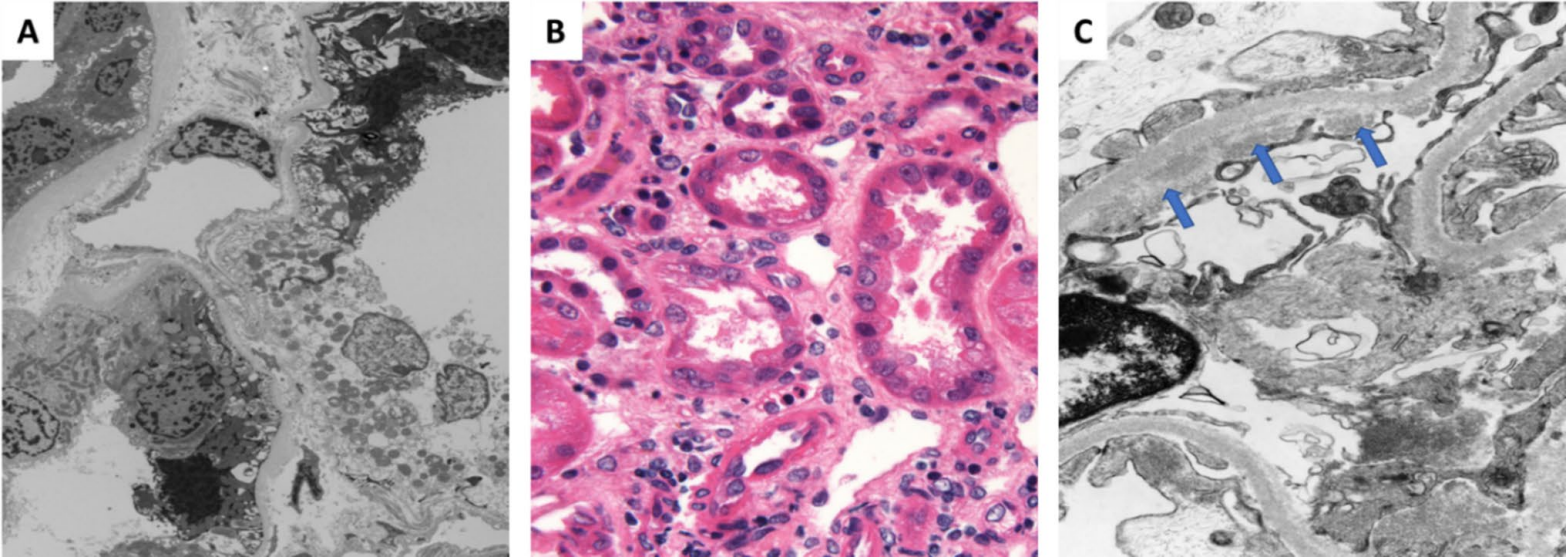
**A** Case 3: Acute tubular injury. Electron microscopy, 1500X magnification. **B** Case 4: Acute tubular necrosis and focal acute interstitial nephritis. Hematoxylin & eosin stain, 400X magnification. **C** Case 4: Subendothelial immune complex deposits (arrow). Electron microscopy, 10000X magnification

A 55-year-old female with stage IV endometrioid uterine carcinoma was started on second-line systemic therapy with pembrolizumab and lenvatinib developed stage 1 AKIN 1 month after treatment. Concurrent medications included omeprazole and antibiotic usage. Her baseline creatinine 0.77 mg/dL subsequently increased to 1.37 mg/dL. She was noted to have 3 + proteinuria. A renal biopsy performed 1 week after initiation of prednisone revealed histologically mild acute tubular injury/acute tubular necrosis and focal and segmental glomerulosclerosis. Acute tubulointerstitial nephritis and thrombotic microangiopathy were not identified. There was minimal tubulointerstitial scarring (Fig. 2). AKI was treated with prednisone 1 mg/kg daily with subsequent taper and full resolution within 2 weeks of treatment. Furthermore, her proteinuria resolved approximately 3 months after initiation of prednisone therapy. Although the FSGS was accompanied by a partial degree of podocyte foot process effacement, this likely reflects partial restoration of podocyte foot process morphology attributed to initiation of steroid therapy prior to the biopsy. Pembrolizumab was discontinued due to renal failure.

#### Case #4

A 69-year-old male with stage IV lung adenocarcinoma with high PD-L1 expression (90%) treated with pembrolizumab monotherapy developed stage 3 AKIN 1 month after treatment. Concomitant notable medications include pantoprazole, NSAID usage, and antibiotics. Her baseline creatinine was 0.88 mg/dL and rose to 7.75 mg/dL. Renal biopsy was suboptimal due to scant sampling of renal cortex. Nonetheless, acute tubular necrosis and focal acute interstitial nephritis were detected, and moreover, electron microscopy study revealed subendothelial immune complex-type deposits in glomeruli which could not be further characterized due to inadequate immunofluorescence microscopy specimen. Due to scant renal cortical sampling, the degree of tubulointerstitial scarring cannot be assessed (Fig. 2). AKI was treated with 1 mg/kg IV methylprednisolone followed by an oral prednisone taper with no significant recovery. The patient remained on hemodialysis. ICI rechallenge was not attempted due to toxicity.

#### Case #5

**Fig. 3:**
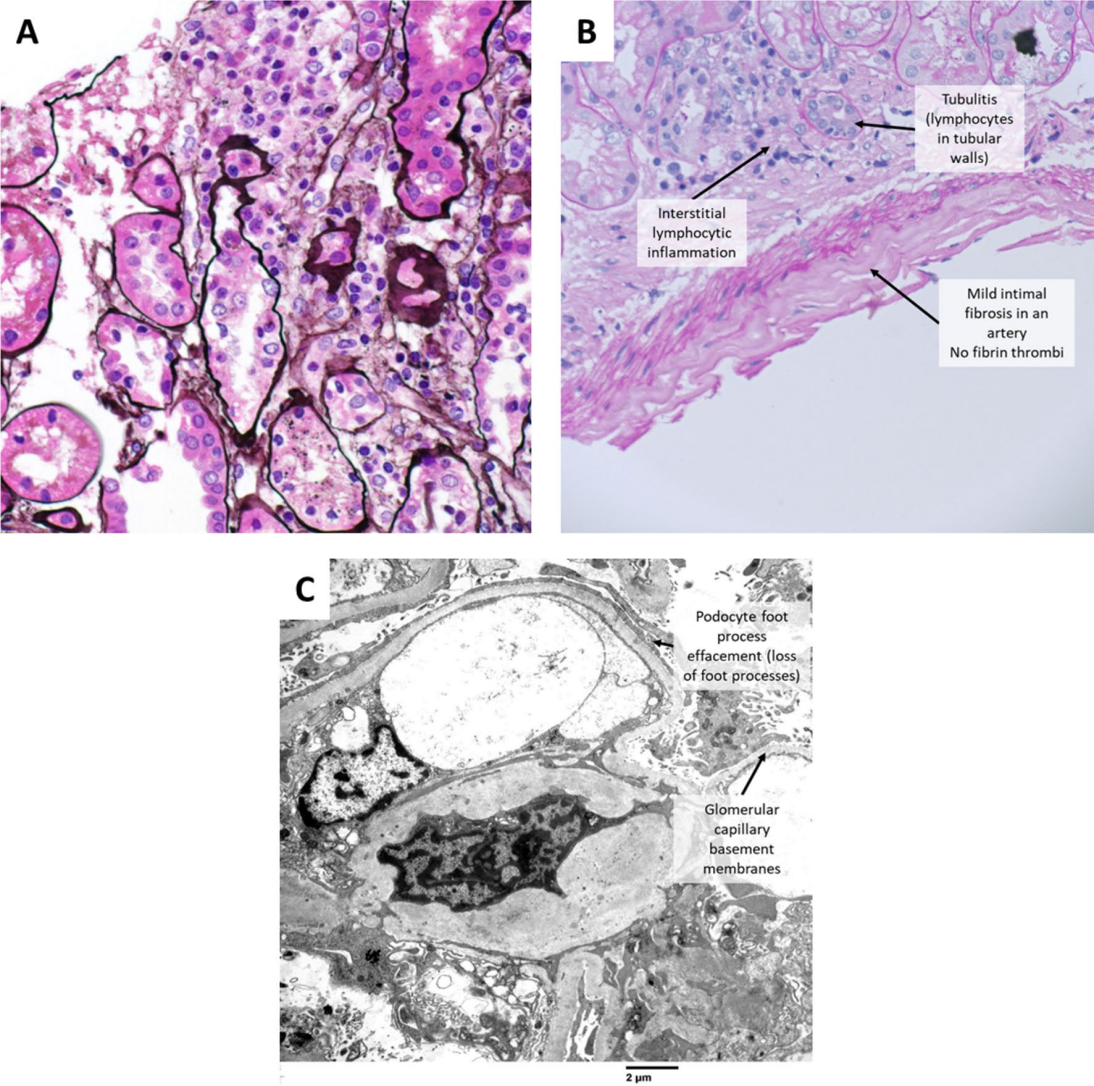
**A** Case 5: Acute tubulointerstitial nephritis. Jones’ silver stain, 400X magnification. **B** Case 5: Mild arteriosclerosis. No thrombotic microangiopathy. PAS stain, 400X magnification **C** Case 6: Extensive podocyte foot process effacement, characteristic of podocytopathy. There are also thickened glomerular capillary basement membranes seen in diabetic glomerulosclerosis. Electron microscopy, 7000X magnification

A 61-year-old female with stage IV lung adenocarcinoma with a high PD-L1 expression (80%) treated with pembrolizumab monotherapy developed stage 3 AKIN 3 months after treatment. Notable concurrent medications include pantoprazole and antibiotic usage. Baseline creatinine was 0.83 mg/dL which subsequently increased to 4.48 mg/dL. Her renal biopsy showed acute tubulointerstitial nephritis, acute tubular necrosis, mild arteriosclerosis, and mild arteriolosclerosis. Glomerular disease and thrombotic microangiopathy were not identified. There was minimal renal tubulointerstitial scarring (Fig. 3). AKI was treated with high-dose prednisone at 1 mg/kg with a subsequent taper with full resolution after 5 months. ICI rechallenge was not attempted due to degree of renal injury.

#### Case #6

A 75-year-old female with stage IV adenocarcinoma of the lung with low PD-L1 expression (1%) and an EGFR Exon 20 insertion and pre-existing stage III chronic renal disease secondary to diabetes mellitus was started on third-line systemic therapy with nivolumab/ipilimumab and developed stage 2 AKIN after 1 cycle of treatment. Concomitant medication use included antibiotics. Baseline creatinine was 1.40 mg/dL, which increased to 3.7 mg/dL. She also developed nephrotic syndrome with urine protein-to-creatinine ratio of more than 28. Her renal biopsy reveals the presence of minimal change disease, mild diabetic glomerulosclerosis, acute tubular injury, mild arteriosclerosis, and mild arteriolosclerosis. There was moderate tubulointerstitial scarring. Acute tubulointerstitial nephritis and thrombotic microangiopathy were not identified. Due to prior steroid-induced psychosis, she was started on tacrolimus therapy and subsequently developed partial response. Her proteinuria improved on spot urine testing from 2000 to 600 mg/dL after 4 weeks of therapy. She did not require hemodialysis, but creatinine remained elevated. She was eventually transferred to hospice care due to declining health.

## Discussion

AKI is increasingly being recognized as a potential complication for patients on ICI therapy [3, 8]. Our case series shows that the clinical manifestations and underlying mechanisms of renal failure in these patients can vary considerably. Some patients may develop a mild rise in creatinine after starting ICIs that may not require any therapeutic intervention or cessation of treatment. Alternatively, others may present with rapidly evolving renal failure requiring the start of renal replacement therapy. Mechanisms of renal injury can include acute interstitial nephritis, acute tubular necrosis, and nephrotic syndrome (such as FSGS or MCD) [9–11]. Risk factors can include pre-existing renal failure due to comorbidities (diabetes mellitus and hypertension) and chemotherapy-induced nephrotoxicity from agents such as cisplatin and pemetrexed. We also show that a significant percentage of patients that develop renal injury while on ICIs are on concomitant medications, specifically PPIs, NSAIDs, and antibiotics. There does not seem to be a predictable time to onset of renal injury after starting ICI therapy as some of our patients developed nephritis after just one cycle of therapy, while others were on therapy for nearly a year before manifesting with AKI. Furthermore, the single patient (case #6) whose serum creatinine remained elevated also had the most abundant pre-existing tubulointerstitial scarring in this case series. Although her negative outcome is confounded by her receiving tacrolimus instead of steroids (due to clinical contraindication), the patient’s underlying chronic kidney disease (i.e., tubulointerstitial scarring) likely plays a significant role. Given the variable presentation of ICI-induced nephritis, treatment to prevent complications may need to be individualized. Moreover, patients with a known history chronic kidney disease should be closely monitored for ICI therapy-related AKI given the potential for worse outcome.

Management of ICI-induced renal failure requires a multidisciplinary approach. In addition to discontinuation or interruption of ICI therapy, first-line therapy with steroids (I.e., prednisone 0.5 to 1 mg/kg/day) remains the mainstay of treatment for most patients with ICI-related renal failure [9, 12, 13]. Exceptions include those who may be unable to tolerate long-term steroid taper due to side effects or underlying comorbidities. Although steroid therapy results in significant clinical recovery in most patients with ICI-induced nephritis, there is still a significant rate of persistent renal failure, and patients may remain on renal replacement therapy long term. In those with nephritis refractory to steroids, renal biopsy may be indicated to confirm that there is no alternative etiology that may be contributing to renal injury. Histopathologic findings may help guide management, which may include cessation of another offending agent, increase in steroid dosing, or additional immunosuppressant therapy (such as tacrolimus, mycophenolate, or infliximab). The use of immunosuppressant agents may help reverse renal injury and help avoid hemodialysis as seen in our patients with ICI-related minimal change disease described above. The prompt recognition and treatment of ICI-related renal injury is essential to achieving optimal recovery of renal function.

Many patients may have an ongoing response to immunotherapy at the time that ICI-related AKI develops. This can cause a conundrum, as several patients will not have alternative effective treatment options available for the treatment of their advanced malignancy. ICI rechallenge may play a role in those who have developed renal failure and subsequently recovered. Given the risk of perpetuating renal damage as well as other IRAEs, a multidisciplinary discussion between oncology and nephrology would be recommended before proceeding with rechallenge in patients with grade 2 or greater nephrotoxicity. The role of continuing low-dose steroids (prednisone 10 mg or less) with rechallenge has been shown to be successful in patients’ other immune adverse events such as ICI-related liver injury and may also be a potential strategy for reducing reaggravation of renal injury in patients undergoing rechallenge [14]. Despite the break in treatment, significant overall survival and real-world progression-free survival benefits were shown in lung cancer patients who developed an IRAE [15, 16]

Of the two patients (cases #3 and #6) who had concomitant proteinuria, the patient (case #3) with FSGS had subsequent resolution of proteinuria after receiving steroid therapy.

For the second patient (case #6), her severe proteinuria was due to MCD. She was started on tacrolimus with partial response (> 50% improvement in proteinuria from > 2000 mg/dL to 600 mg/dl on spot urine protein testing), but no significant improvement in creatinine.

It was unfortunate that the patient in case #6 with proteinuria, who fell within the small subset (approximately 10%) of patients with MCD, did not have a robust response to tacrolimus therapy. In this case series, these two proteinuric patients and microanatomic glomerular lesions of FSGS and MCD are best classified as paraneoplastic glomerular diseases [17].

Our aim in gathering CGP-based alterations was to check for possible associations with the occurrence of IRAEs. Evaluation of tumor tissue on Case #6 revealed an exon 20 insertion which can be treated with FDA approved target therapies. However, this patient had severe complications from ICI-related renal injury that prevented the patient from starting targeted therapy. This highlights the importance of utilizing CGP results for optimal therapy selection. Remaining mutational analysis did show multiple patients with TP53 and NF1 mutations, but these mutations are extremely common across the spectrum of malignancies and are unlikely to be predictive of ICI-related toxicities. There was no other comutations noted on CGP that appeared to correlate to the development of ICI-related AKI. PD-L1 expression on tumor samples was high in two out of six patients, but low in the remaining four patients. TMB was only evaluable in three patients, none of which were TMB high. All six patients were noted to be microsatellite stable. Given the limited number of patients analyzed in our study, we were not able to significantly associate any CGP mutations, level of PD-L1 expression, TMB status, or MSS status with ICI-related renal injury. Germline analysis may be of benefit in determining patients predisposed to AKI from ICIs [18]. In addition, PD-L1 testing on renal biopsy specimens may be of clinical utility [19, 20].

In conclusion, ICI-related AKI should be recognized as a potential side effect of both single-agent and dual-checkpoint inhibitor therapy. A high degree of clinical suspicion is required to ensure early diagnosis and treatment to help improve outcomes. In addition to renal tubulointerstitial diseases, there may also be concomitant glomerular and extraglomerular vascular diseases. However, thrombotic microangiopathies are not detected in our small case. Monitoring of blood work including BUN/Cr and urine studies to detect evolving proteinuria can help determine which patients may be developing this rare complication. A renal biopsy can be a useful tool in determining the mechanism of renal failure and can help guide treatment [20–22]. Escalation of treatment to higher doses of steroids or immunosuppressants should be initiated within 1 week in those patients who may be refractory to initial therapy [12]. Limitations of this study include the small sample size and its retrospective nature. Larger studies are needed with prospective data collection and a more uniform criteria for collection of renal biopsies. More researches into potential predictive biomarkers and studies exploring the mechanistic pathways of ICI-related AKI are needed to determine which patients may be at higher risk for ICI-related renal injury.

## Data Availability

No datasets were generated or analyzed during the current study.
